# Molecular Characterization of Noroviruses and HBGA from Infected Quilombola Children in Espirito Santo State, Brazil

**DOI:** 10.1371/journal.pone.0069348

**Published:** 2013-07-22

**Authors:** Fernando Vicentini, Wilson Denadai, Yohanna Mayelle Gomes, Tatiana L. Rose, Mônica S. R. Ferreira, Beatrice Le Moullac-Vaidye, Jacques Le Pendu, José Paulo Gagliardi Leite, Marize Pereira Miagostovich, Liliana Cruz Spano

**Affiliations:** 1 Departamento de Ciências da Saúde, Universidade Federal do Espírito Santo, São Mateus, Espírito Santo, Brazil; 2 Programa de Pós-Graduação em Doenças Infecciosas, Universidade Federal do Espírito Santo, Vitória. Brazil; 3 Departamento de Patologia, Universidade Federal do Espírito Santo, Vitória, Espírito Santo, Brazil; 4 Laboratorio de Virologia Comparada e Ambiental, Instituto Oswaldo Cruz, Fundação Oswaldo Cruz, Rio de Janeiro, Brazil; 5 Institut National de la Santé et de la Recherche Médicale, Nantes, France; 6 Centre de Recherche en Cancérologie, Nantes, France; 7 Université de Nantes, Nantes, France; Northeast Agricultural University, China

## Abstract

Noroviruses (NoV) are the main etiological agents of gastroenteritis outbreaks worldwide and susceptibility to NoV infection has been related to the histo-blood group antigen (HBGA). This study aimed to determine the prevalence of NoV strains and to evaluate the HBGA phenotype and genotype of children from semi-isolated Quilombola communities, descendents of black slaves in Brazil. A total of 397 children up to eleven years old, with and without diarrhea, from Quilombola Communities in the Espirito Santo State, Brazil, were investigated for the presence of NoV from August 2007 to September 2009. Feces were collected from all the children, and blood from the NoV positive children. NoV was screened by reverse transcription-PCR with primers for the RNA-dependent RNA polymerase region; genogroup was determined by PCR with primers for the C and D regions and genotyped by sequencing. HBGA phenotype was performed by gel-spinning and *FUT2* and *FUT3* were analyzed by PCR or sequencing analysis. NoV were detected in 9.2% (12/131) of diarrheic and 1.5% (4/266) of non-diarrheic children (p<0.05, Fisher’s exact test). GI and GII genogroups were present in 12.5% and 87.5% of the samples, respectively. The following genotypes were characterized: GII.4 (25%), GII.12 (25%), GII.6 (12.5%) and GI.1 (6.3%), GI.3 (12.5%) and GI.4 (6.3%). Children infected with NoV showed the A (n = 6), O (n = 6), and B (n = 2) HBGA phenotypes, and 13 of them were classified as secretors (*Se*) and one as a non secretor (*se*). Mutations of *Se* ^40^, ^171,216,357,428,739,960^ were found for the *FUT2* gene and mutations of *Le* ^59, 202, 314^ for the *FUT3* gene. The only *se* child was infected by NoV GI, whereas the *Se* children were indiscriminately infected by GI or GII. This study showed rates of NoV infection in symptomatic and asymptomatic Quilombola children consistent with other studies. However, children under 12 months were seven times more affected than those between 1 and 5 years old. GII.12 was as frequent as GII.4 and GI.1 and GI.4 were described for the first time in Brazil. Owing to the small number of cases studied, no clear pattern of susceptibility and/or HBGA resistance could be inferred.

## Introduction

Gastroenteritis of infectious etiology is still an important cause of morbidity in the human population worldwide [1]. Diarrhea and vomiting associated with a lack of access to primary health care and supportive treatment for dehydration, can lead to serious clinical consequences, particularly in developing countries [2], [3]. Noroviruses (NoVs) are the main viral agents in acute diarrhea outbreaks and sporadic cases for all age groups worldwide, except for rotaviruses in children under three years old, leading to more than 1 million hospitalizations annually [1], [3]. However, this scenario is changing in countries, including Brazil [4] that have adopted the use of rotavirus vaccines tending to pass all age groups be headed by NoV. In addition, asymptomatic excretion is reported in healthy individuals, favoring virus transmission [1], [2], [3], [5].

Human NoVs belong to the *Caliciviridae* family, *Norovirus* genus and are classified into three genogroups: GI, GII, and GIV. Differences in the sequence of the major viral capsid proteins (VP1) allow further classification into eight GI and twenty-one GII genotypes and one GIV genotype [6]. GII.4 is considered the most prevalent genotype worldwide [1], [7].

At present, there is no *in vitro* replication system available for human NoVs. Nevertheless, the expression of recombinant VP1 allows the *in vitro* reconstruction of VLPs (virus-like particles), morphologically and antigenically similar to the wild virions [8] that show bind to molecules characterized as histo-blood group antigens (HBGA) [9], [10], [11], [12], [13], [14]. HBGAs are oligosaccharides synthesized by the stepwise addition of monosaccharides onto glycan precursors via the glycosyltransferases FUT2, FUT3 and A/B of the ABO and Lewis blood group systems. The FUT2 enzyme adds a fucose, in the α1,2 linkage, onto a galactose of the precursor, generating the H antigens. The FUT3 enzyme adds a fucose residue, in the α1,4 (or 1,3) position, onto the N-acetylglucosamine of the precursor, generating the Lewis a (Le^a^) or the Lewis b (Le^b^) antigens when combined with the α1,2 fucose residue. The A or B enzymes catalyze the addition of an N-acetylgalactosamine or a galactose onto the H antigens, giving rise to the A and B antigens, respectively. The *FUT2*, *FUT3* and *ABO* genes possess functional alleles encoding the FUT2, FUT3, A and B enzymes respectively. These *FUT2*, *FUT3* and *ABO* genes also possess null alleles which are unable to generate active enzymes. Individuals who have the H antigen in their epithelial tissues and secretions are called secretors. In contrast, individuals who inherited two *FUT2* null alleles are devoid of H, Le^b^, A and B antigens and are called non-secretors. Similarly, *FUT3* null homozygotes lack the Le^a^ and Le^b^ antigens and homozygotes for O alleles (null alleles of the *ABO* gene), are devoid of either A and B antigens and are therefore of the blood group O [15].

Approximately 80% of the human population has a secretor phenotype (*Se*) and is considered to be susceptible to NoV infections by the majority of strains with a variation depending on the ABO phenotype and on the ability of strains to recognize the A, B, H or Le^b^ antigens. The remaining 20% of the population with the non secretor phenotype (*se*) is considered to be naturally resistant to most NoV strains [16], [17], [18], [19]. However, VLPs from some GI and GII strains have been reported to bind to carbohydrates such as Le^a^ or Le^x^ mainly present in non-secretors. Accordingly, non-secretor individuals are occasionally infected by NoVs [14], [20], [21], [22], [23].

Although HBGA polymorphisms representing populations from the five continents have already been characterized, the populations studied hitherto in South America have been restricted to the Colombian Amerindians, Maya, Suruí, Karitiana and Pima Indians [24]. This is the first study involving a black population from Southeastern Brazil, and moreover, infected with NoV. This population consists of descendants of former African slaves, who live in semi-isolated communities, called “Quilombola Communities” and the people living there are known as “quilombolas” [25]. Even today, in the 21^st^ century, they are still underserved communities with poor sanitary conditions, conducive to gastrointestinal diseases.

The aims of this study were: i) to determine the prevalence and genotypes of NoV among children up to 11 years old, with and without diarrhea, who are residents in the Quilombola Communities in the Espírito Santo State, Southeastern Brazil and; ii) to characterize the HBGA phenotypes and genotypes of the infected children to elucidate their susceptibility to NoV infection.

## Materials and Methods

### Study Design and Site Description

This is a descriptive study with Quilombola children up to 11 years old, who are descendants of slaves and who live in Quilombola Communities located in the semi-isolated rural areas, known as North Sapê, in the North of Espírito Santo State, Southeastern Brazil. Today there are 30 communities in the North Sapê region with an estimated population of more than 3,600 people, 950 residences and numerous farms dedicated to the cassava plant. They are between 6.21 mi (10 km) and 18.64 mi (30 km) from urban centers and from each other, and are connected by dirt roads of very difficult access.

This study was approved by the Ethics Research Committee of the Centro de Ciências da Saúde, Universidade Federal do Espírito Santo (002A/08) and Statement of Consent was obtained from the guardians and from the children themselves, whenever appropriate.

### Fecal and Blood Specimens

A total of 397 fecal specimens were obtained, 131 (33%) from children with diarrhea (symptomatic) and the remaining 266 (67%) from children without diarrhea (asymptomatic), matched by community, between August 2007 and September 2009. To carry out the HBGA studies, blood samples (3 ml) were collected by venipuncture in tubes containing ethylenediamine tetraacetic acid (EDTA) as anticoagulant from children who were positive for NoV. Blood samples were immediately analyzed for phenotype as described below; leukocytes were obtained by centrifugation at 2,000×g for 15 min and stored at −20°C for further analysis. [26]. Feces and peripheral blood leukocytes were frozen at −20°C for nucleic acid extraction.

### Noroviruses Detection and Genogroup and Genotype

Viral nucleic acid was extracted from a 10% fecal suspension in Tris-calcium by using a guanidine isothiocyanate and silica method, as previously described [27]. Complementary DNA (cDNA) was obtained in a reverse transcription reaction using the 20 mU random primer pd(N)_6_™ (Amersham Bioscience, UK) and the SuperscriptII™ reverse transcriptase (Invitrogen, USA) [28].

NoV detection was performed through PCR using two sets of primers which hybridize to the *RdRp* gene (RNA-dependent RNA polymerase - ORF1 viral genome), MON 431/433 and MON 432/434, as described by Beuret et al. (2002) [28] and following the protocols proposed by Victoria et al. (2007) [29]. The genogroup determination was conducted with two PCR mixtures containing a pair of primers for GI (SRI-1/SRI-2) [28] and GII (MON381/MON383) [30] and the primers (G1SKF/G1SKR and G2SKF/G2SKR) [31], and (Cap A, B1, B2/Cap C, D1, D3) [32], specific for the C and D region of the VP1 capsid gene (ORF2), respectively. All primers used are described in Table 1.

**Table 1:** Nucleotide sequence of the primers used in amplification and sequencing of the norovirus genome.

Primer	Sequence 5′ to 3′	ORF/Region	Position	Fragment	Reference
MON 431	TGG ACI AGR GGI CCY AAY CA	ORF1/B	5093	213 pb	30
MON 433	GAA YCT CAT CCA YCT GAA CAT	ORF1/B	5305		
MON 432	TGG ACI CGY GGI CCY AAY CA	ORF1/B	5093	213 pb	30
MON 434	GAA SCG CAT CCA RCG GAA CAT	ORF1/B	5305		
GI SRI 2	AAA TGA TGA TGG CGT CTA AG	ORF2/C	5344	222 pb	28
GI SRI 3	AAA AYR TCA CCG GGK GTA T	ORF2/C	5566		
GII MON381	CCAGAATGTACAATGGTTATGC	ORF2/C	5362	322 pb	30
GII MON383	CAAGAGACTGTGAAGACATCATC	ORF2/C	5683		
GI Cap B1	TAT GTT GAC CCT GAT AC	ORF2/D	6738	177 bp	32
GI Cap B2	TAT GTI GAY CCW GAC AC	ORF2/D	6738		
GI Cap A	GGC WGT TCC CAC AGG CTT	ORF2/D	6914		
GII Cap D1	TGT CTR STC CCC CAG GAA TG	ORF2/D	6432	253 bp	32
GII Cap D3	TGY CTY ITI CCH CAR GAA TGG	ORF2/D	6432		
GII Cap C	C CCT TYC CAK WTC CCA YGG	ORF2/D	6684		
GI G1SK F	CTG CCC GAA TTY GTA AAT GA	ORF2/C	5342	329 bp	31
GI G1SK R	CCA ACC CAR CCA TTR TAC T	ORF2/C	5671		
GII G2SK F	CNT GGG AGG GCG ATC GCA A	ORF2/C	5058	343 bp	31
GII G2SK R	CCR CCN GCA TRH CCR TTR TAC AT	ORF2/C	5401		

The positions of the primers are relative to the entire genome of the GI Norwalk M87661 and GII Lordsdale X86557 strains.

NoVs genotyping was determined by partial sequencing of PCR amplicons obtained with primers for ORF-2 (G1SKF/G1SKR and G2SKF/G2SKR for the C region and Cap A, B1, B2/Cap C, D1, D3 for the D region of the VP1 gene) [31], [32] (Table 1). Amplicon purification was performed using the QIAquick® kit (Qiagen) and sequencing using the commercial kit BigDye® Terminator v3.1 Cycle Sequencing Kit (Applied Biosystems, CA, USA). Sequences were aligned using the BioEdit Sequence® Alignment Editor version 7.0.9.0, deposited in the GenBank database and then compared with sequences of Brazilian and reference samples of each genotype. A phylogenetic tree was constructed by the Neighbor-joining method using the MEGA program version 5.10, and the genetic distance was calculated with the Kimura 2 parameter model, using 2,000 pseudo-replicates.

Nucleotide sequences obtained in this study were submitted to the National Center for Biotechnology Information (Gen Bank, http://www.ncbi.nlm.nih.gov/) and received the following accession numbers: region C, Q51-JX893035, Q220-JX898882, Q230-JX898883, Q225-JX898884, Q254-JX898885, Q352-JX898886, Q322-JX898887, Q323-JX898888, Q325-JX898889, Q151-JX898890. Region D, Q151-KC113519, Sape95-KC113520, Q278-KC113521, Q225-KC113522, Q326-KC113523 and Q323-KC113524.

### Histo-Blood Group Antigen

#### Phenotypic analysis

The characterization of the blood groups was carried out with the peripheral blood samples from the NoV positive children by: (i) the tube agglutination technique with anti-A, anti-B, anti-AB and anti-D (DiaClon™, Diamed, Br, BIO RAD) monoclonal antibodies (MAbs), and (ii) the Lewis antigens detection system and the secretor status categorization by gel-centrifugation was performed with capture MAbs anti-Le^a^ or anti-Le^b^ (DiaClon™, Diamed, Br, BIO RAD) in two different reactions, according to the manufacturer’s instructions.

#### Genotypic Analysis of the *FUT2* gene (secretor)

Genotype analysis was carried out with DNA extracted from peripheral blood leukocytes by treatment with 1.6 M sucrose and 10 mg/ml proteinase K [33]. Inactivating mutations of *FUT2* gene were screened by: (i) PCR for the most common mutation G428A with specific primers for the mutation *Se* antisense (as) combined with *Se* 1s sense (s) for the wild-type allele and PCR with primers for the mutant gene *se* with *Se*2s, as previously described [26] (Table 2) and; (ii) gene sequencing of exon 2, using a set of primers designed for this study from the reference sequence available in GenBank (NCBI). The primers were targeted to amplify the entire exon, from the position –61F, up to +98 R, as follows:

**Table 2:** Set of primers used for detecting mutations in FUT3.

Primer	Object	Sequence 5′ to 3′	Fragment	Reference
hGH1	Control	GCCTTCCCAACCATTCCCTT	428 pb	34
hGH2	Control	TCACGGATTTCTGTTGTGTTTC		
Se-as	G428A	GGCTGCCTCTGGCTTAAAG	520 pb	26
Se-s selvage	G428A	GCTACCCCTGCTCCTGG		
Se-s mutant	G428A	CGGCTACCCCTGCTCCTA		
III-55as	Reverse	TTCTGGAGGGGAGAGGCT		
III-48s selvage	T59G	CGCTGTCTGGCCGCACT	1186 pb	26
III-47s mutant	T59G	GCTGTCTGGCCGCACGG		
III-50s selvage	T202C	CCCTCCTGATCCTGCTATG	1045 pb	26
III-49s mutant	T202C	ACCCTCCTGATCCTGCTAC		
III-52s selvage	C314T	GTACCCACAGGCAGACACG	932 pb	26
III-51s mutant	C314T	TGTACCCACAGGCAGACAT		
III-54s selvage	T1067A	CCAGACGGTGCGCAGCAT	180 pb	26
III-53s mutant	T1067A	CCAGACGGTGCGCAGCAA		

Forward primers: (i) position –61/hFut2 –61F (TGAGGTGCCTGCCCAACC ACTCTGT); (ii) position 1/hFut2 met (ATGCTGGTCGTTCAGA TGCCT); (iii) position 489/hFut2.3 (GATCCTCCAGGA GTTCACCCTGCA); (iv) position 699/hFut2 699 F (CGCTACAG CTCCCTCATCTTCGTG).

Reverse primers: (i) position +98/hFut2+98 R (AGAGATGGGTCCTGCTCA TGGAAC); (ii) position 1009/hFut2 stop (TGTCCCCCTTACTCAAGCACTAA); (iii) position 569/hFut2 564 R (AGCCGGCCGGGCACCTTTGTAGGGGTCCAT); (iv) position 281/hFut2 267 R (TCATCTTGGCCAG GGCGTACAGTGT).

A 50 µl final reaction mixture contained 0.2 mM dNTP, 1.5 mM MgCl_2_, 0.2 µM of each primer, 1 unit of Platinum Taq DNA polymerase (Invitrogen®, Carlsbad, CA, USA), 1× PCR buffer and 1 µl of DNA under the following conditions: initial denaturation at 95°C for 2 min followed by 25 cycles at 95°C for 45 s, 60°C for 45 s and 72°C for 75 s, and a final extension at 72°C for 5 min. The gene for human growth hormone (HGH) was used as the internal control of the reaction [34].

For sequencing, the PCR products were purified with ExoSAP-IT™ (GE/USB). The thermal cycle was 37°C and 80°C for 15 min. each. Sequencing was performed with the BigDye® Terminator v3.1 Cycle Sequencing Kit (Applied Biosystems, CA, USA). Sequences were aligned using the BioEdit Sequence Alignment Editor version 7.0.9.0.

#### Genotypic Analysis of the *FUT3* gene (Lewis)

The presence of four major human *FUT3* mutations T59G, T202C, C314T and T1067A was investigated [26]. Two PCRs were performed for each mutation with a set of primers containing III-55 antisense and (i) the initiator for the wild-type allele and (ii) the initiator for the mutant allele (Table 2). One microliter of DNA extracted was added to a 50 µl final reaction mixture containing 1.5 mM MgCl_2_, 200 µM of each dNTP, 0.2 µM of each primer and 1 unit of GoTaq® DNA polymerase (Promega, Madison, USA). Amplification was performed under the following conditions: initial denaturation at 95°C for 2 min followed by 25 cycles at 95°C for 45 sec, 60°C for 45 s and 72°C for 75 s, and final extension at 72°C for 5 min. Similarly to the previous item, an internal control reaction was performed.

### Statistical Analyses

The two sided Fisher’s exact test was performed for comparisons between groups using the GraphPad program version 5.0a, and p values below 0.05 were considered significant.

## Results

### Detection and Molecular Characterization of Noroviruses

Noroviruses were detected in 4% (16/397) of the fecal specimens obtained from Quilombola children, of whom 131 (33%) were diarrheic (symptomatic) and 266 (67%) were without diarrhea (asymptomatic). Seventy percent of the cases occurred between March and May 2009. The ratio of NoVs positivity among symptomatic and asymptomatic children was 9.2% (12/131) and 1.5% (4/266), respectively. Children up to 12 months old corresponded to 9.6% (38/397) of the specimens and represented 50% (8/16) of the NoVs positive cases and 87.5% (7/8) of these occurred in symptomatic children. The ratio of positive cases among children up to 12 months old was 21% (8/38), whereas in children between 1 and 5 years old it was 3.2% (5/155). The difference in NoV prevalence between children up to 12 months old and older children was statistically significant (p<0.002, Fisher’s exact test).

Among the genogroups characterized, GII represented 87.5% (14/16) and GI 18.5% (3/16) of the cases; one was a GI and GII mixed infection.

**Figure 1:**
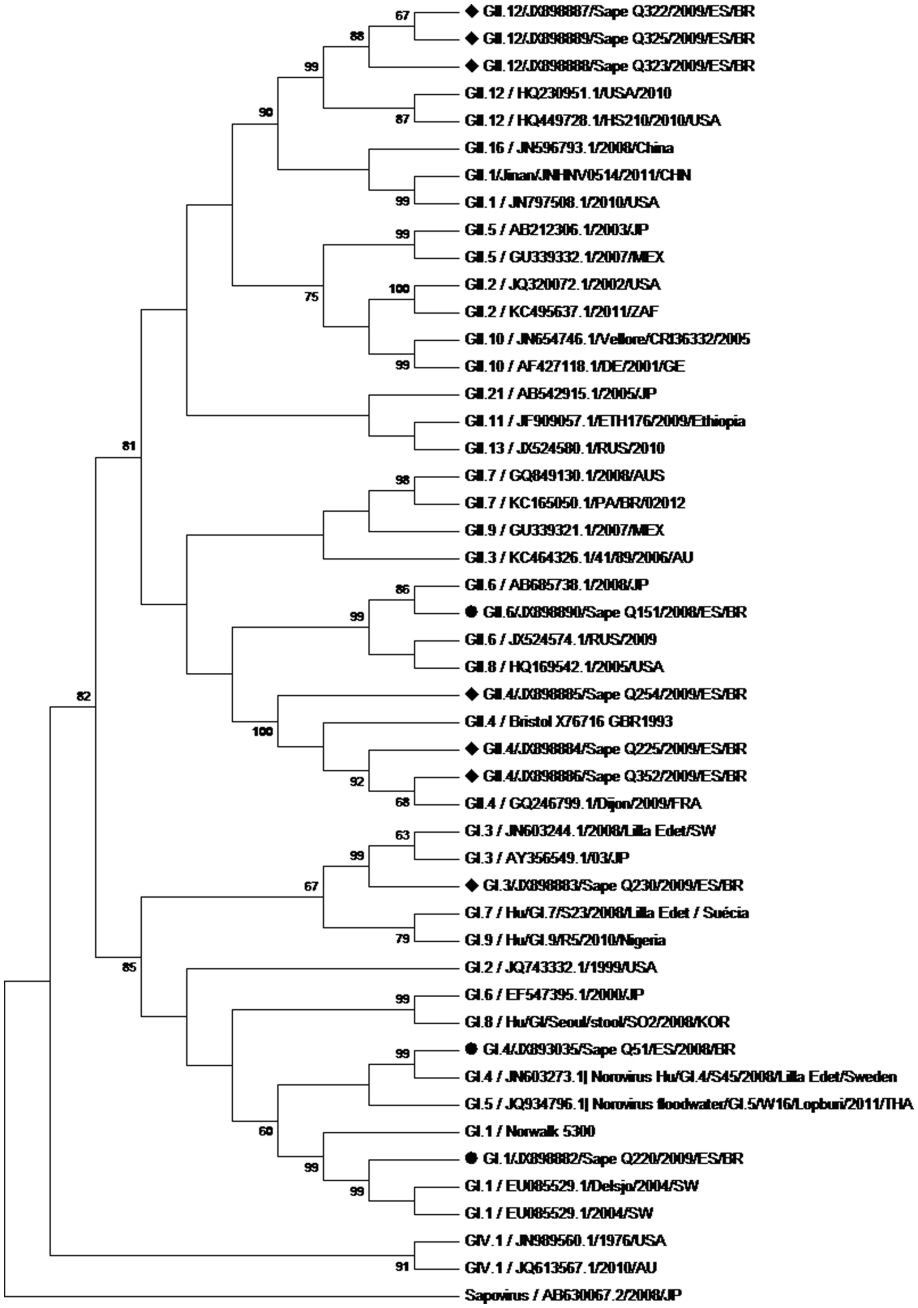
Phylogenetic tree of C region. Phylogenetic tree constructed by the Neighbor-joining method based on the partial nucleotide sequence of the norovirus VP1 capsid protein gene C region. Highlighted strains identified by “Sapê”, originated in fecal samples from “Quilombola” children and were analyzed along with the prototype norovirus genotypes of GI, GII and GIV taken from the “GenBank.” All strains were identified by genotype/accession number/year/origin. Sapovirus/AB630067.2/2008/JP was added as a reference group. Values of “Bootstrap (2,000 replicates) are shown at the junction of the branches. • Samples Q51, Q151 and Q220 belong to another study site near the Quilombola communities. ♦ Samples of this study.

**Figure 2:**
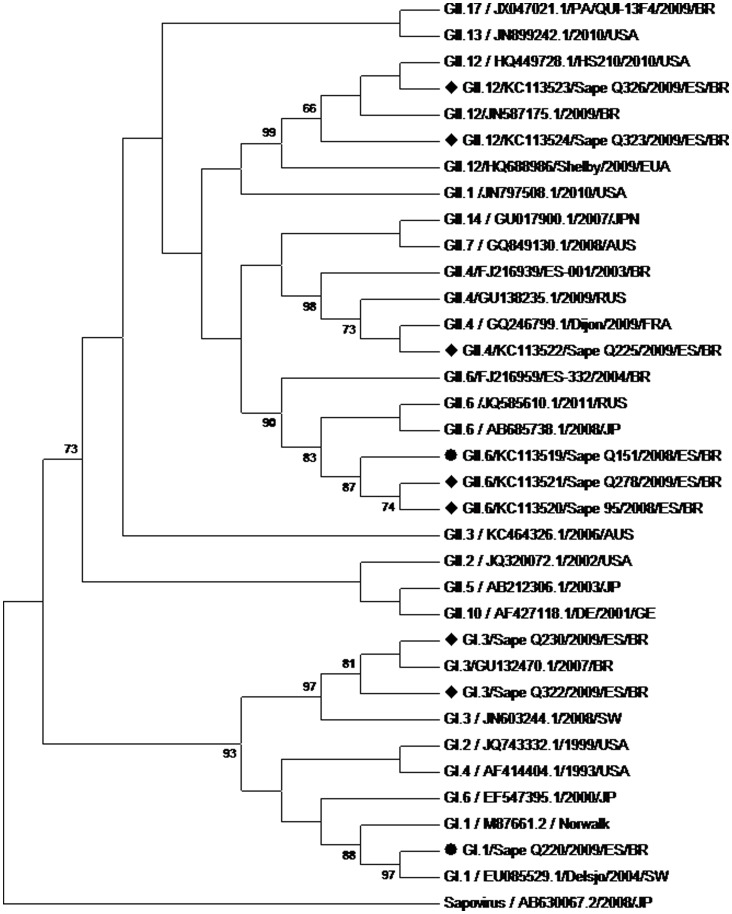
Phylogenetic tree of D region. Phylogenetic tree constructed by the Neighbor-joining method based on the partial nucleotide sequence of the Norovirus VP1 protein gene D region. Highlighted strains identified by “Sapê”, originated in fecal samples from “Quilombola” children and were analyzed along with prototype norovirus genotypes of GI and GII taken from the “GenBank.” All strains were identified by genotype/accession number/year/origin. Sapovirus/AB630067.2/2008/JP was added as a reference group. Values of “Bootstrap (2,000 replicates) are shown at the junction of the branches. • Samples Q51 and Q220 belong to another study site near the Quilombola communities. ♦ Samples of this study.

Twelve strains were genotyped by means of partial sequence analysis of ORF2 (regions C and D), evidencing GII.4 (n = 4), GII.12 (n = 4), GII.6 (n = 2), and GI.3 (n = 2) genotypes (Table 3, Figures 1 and 2). GII.6 was detected exclusively among asymptomatic children, while the other genotypes were found only among symptomatic cases. The four strains that could not be genotyped were three GIIs (two from symptomatic cases and one from an asymptomatic case), and one GI (from an asymptomatic case). NoV GI.1, GI.4 and GII.6 (Figures 1 and 2) were also detected in an urban community, consisting of predominantly black people, near the traditional Quilombola Communities (unpublished results).

**Table 3:** Genotype of NoVs and HBGA profile of infected individuals.

Sample	NoV Genotype	Diarrhoea	ABO/Rh	Lewis	Secretor status	PCR mut	PCR mut
						G428A	Lewis
Sapê Q254^1^	GII.4	S	A+	a-b+	*Se*	*SeSe*	*LeLe*
Sapê Q225	GII.4	S	O+	a-b+	*Se*	*SeSe*	*Lele^59^*
Sapê Q352	GII.4	S	ND	ND	ND	ND	ND
Sapê Q323	GII.12	S	A+	a-b+	*Se*	*SeSe*	*LeLe*
Sapê Q325	GII.12	S	A+	a-b+	*Se*	*SeSe*	*LeLe*
Sapê Q326	GII.12	S	A+	a-b+	*Se*	*SeSe*	*Lele^59^*
Sapê Q322^2^	GI. 3+ GII.12	S	O+	a-b+	*Se*	*SeSe*	*Lele ^202,314^*
Sapê Q230	GI.3	S	O+	a-b-	*Se*	*SeSe*	*Lele^59^*
Sapê Q229	GII.?	S	O+	a-b+	*Se*	*SeSe*	*LeLe*
Sapê Q174	GII.?	S	B+	a-b-	*Se*	*SeSe*	*Lele^59^*
Sapê Q304^2^	GII.4	S	B+	a-b+	*Se*	*SeSe*	*Lele ^202,314^*
Sapê Q028	GII.?	S	A+	a-b+	*Se*	*SeSe*	*LeLe*
Sapê Q278	GII.6	N	A+	a-b-	*Se*	*SeSe*	*Lele^59^*
Sapê 95	GII.6	N	O+	a-b+	*Se*	*SeSe*	*Lele ^202,314^*
Sapê Q355	GII.?	N	A+	a-b+	*Se*	*Sese^428^*	*LeLe*
Sapê Q329	GI.?	N	O+	a+b-	*se*	*se^428^se^428^*	*Lele ^202,314^*
Q51*	GI.4	S	ND	ND	ND	ND	ND
Q151*	GII.6	S	ND	ND	ND	ND	ND
Q220*	GI.1	N	ND	ND	ND	ND	ND

1– Q254 is the same child as Q028 (specimens obtained 6 months apart). 2– Sequences smaller than 200 bp, sorted by Blast/NCBI.

* Samples obtained at the same period of the study from children of neighboring communities, not characterized as Quilombola. Se – secretor/se – non secretor/Le – Lewis wild-type allele/le – Lewis null allele. N – asymptomatic and S – symptomatic.

HBGA was determined for 14 out of the 16 children infected with NoV, six belonged to blood group A, six to O and two to B (Table 3); 13 children were characterized as *Se* and one as *se*. The *Se* children were infected by GI and GII NoV strains, while the only *se* was infected by a GI strain. The frequency of *se* individuals in this population is close to 0.2 (data not shown) and individuals of this phenotype are under-represented among infected children (1/14). All three children infected by GI belonged to the O blood group, while those infected by GII were equally distributed between A and O blood groups. Here again, this difference did not reach statistical significance owing to the small number of cases.

### Characterization of the Polymorphisms of the *FUT2* and *FUT3* Genes

The full coding sequence of the *FUT2* gene revealed several mutations in the black population studied, at the 40, 171, 216, 357, 428, 739 and 960 positions (Table 4). The inactivating mutation G428A was in a homozygous state in the only child with *se* phenotype.

**Table 4:** Mutations found in exon 2 of the FUT2 gene of people from different continents and of the population studied.

BGMUT ID	Geographic origin	40	171	216	357	428	739	960	Reference
301	reference	A	A	C	C	G	G	A	1*
313, 317, 1018	Africa	G	G	T	T	A	A	G	1*
329, 801, 949	Asia		G	T	T	A	A	G	1*
1001, 1004, 1132	Europe		G	T	T	A	A	G	1*
	Brazil	G	G	T	T	A	A	G	2*

1* – Adapted from BGMUT [50].

2* – This study.

For the *FUT3* gene, five children were homozygotes for the wild-type alleles. The mutations *Le* ^59^ (n = 5) and *Le* ^202,314^ (n = 4) were always detected in the heterozygous state, both in those phenotypically characterized as Lewis positive and those characterized as negative, suggesting that other mutations could account for the Le (a-b-) phenotype in this population (Table 3).

## Discussion

NoVs were found in the Quilombola children at rates consistent with other studies addressing community infection by NoVs [35], [36], [37]. Despite this, the rate of NoVs among the symptomatic and asymptomatic children was relatively high, since the studied communities are rural and sparsely populated, which could compromise the transmission of virus particles. Although the positive NoVs cases were concentrated between March and May 2009, the great diversity of the genotypes coupled with the small number of cases in each community at each collection date does not allow this result to be characterized as an outbreak.

NoV infection is known to occur in individuals of all ages due to its vast diversity of genotypes, its infectivity, the high rates of mutation and recombination which together lead to antigenic varieties and escape from the host immune system [29], [38], [39], [40], [41]. However, in our study, most cases of symptomatic NoV infection occurred among children up to 12 months old. Although this age group corresponded to only 9.6% of the total of samples collected, it had a higher prevalence compared to the prevalence found in the 1 to 5 year-old children, suggesting there was either a greater susceptibility of very young children or early immunization in this type of community. Ferreira et al. (2012) [42] also showed a higher prevalence of NoVs in the youngest children, but in children older than those in this present study.

This study of NoVs in 30 Quilombola communities of Southeastern Brazil was conducted in parallel to the recently published study carried out in a similar community in the North of Brazil [25]. These regions are approximately 2,300 km apart from each other. In contrast to our results, the study in the North showed twice the number of NoV cases in children with diarrhea but did not find the virus in any of the asymptomatic children [25]. This study complements earlier studies with hospitalized patients or outpatients at emergency rooms that involved patients with a more severe clinical profile [3]. Ferreira et al. (2012) [42] in a 15 year retrospective study of outbreaks or sporadic cases of gastroenteritis, found NoVs in 28.8% of all cases.

Most of the NoV strains characterized in this study belonged to GII, in agreement with other studies that show GII as the most prevalent in the world, whereas GI is sporadically present at high prevalence [1], [7], [35]. However, a third the cases here consisted of GI, a rate that can be considered relatively high for Brazil since previous studies have described lower frequencies [3], [42], [43] as well as the Quilombola study in the North [25], although Soares et al. (2007) [44] described a GI prevalence of 47.6% in Brazil.

This study shows the first description for GI.1 and GI.4 in Brazil among the three different GI genotypes detected (GI.1, GI.3 and GI.4), which represents a relatively high diversity. Studies in others countries have also shown high diversity of GI types, albeit with a low frequency for each [43], [45]. Generally, the diversity observed for GI and GII was interesting because they were found in semi-isolated communities. A broad genetic diversity was also observed in the Quilombola children in the North of Brazil [25].

The GII.12 strains align very closely to those referred to as “post-2009”, when the rise of a new NoV GII.12 recombinant in the U.S. winter 2009/2010 was described [40]. Interestingly, stools of the present study were collected six months before the North American samples (March 2009), suggesting that the new GII.12 recombinant probably circulated in Brazil concomitantly to the U.S. Nevertheless, one should take into account that the sequence alignment was made on the basis of the C and D regions of VP1 gene, requiring the sequence of B region and its interface with C region to prove a recombinant phenomenon, as recently described by Fumian et al. (2012) [39].

In the last decade the most prevalent genotype, in people of all ages worldwide has been the GII.4 [1], [43]. The GII.4 strains observed were similar to the 2006b variant, shown in Figure 1 by the prototype GQ246799.1/Dijon/2009/FRA. This variant has been detected in at least seven other states in Brazil [43]. In terms of its epidemiological impact, worldwide since 1995, this genotype has been widely studied in order to understand its mechanisms of viral evolution and escape from the immune system as well as its ability to recognize HBGAs [38], [46], [47], [48], [49]. In the present study, we observed that it was not as overwhelmingly represented as previously reported but was similar in prevalence to GII.12. This suggests that GII.4 may be in the process of being replaced by the new GII.12 recombinant as the dominant circulating strain.

To assess if there was any parasite-host relationship typical for Brazil, we characterized the HBGA phenotype and genotype of the Quilombola children infected with NoVs, as representative of people of African descendant in Brazil. They correspond to 50% of the population, according to the Brazilian Institute of Geography and Statistics (IBGE 2011).

Here, no significant association between infection and ABO, secretor, or Lewis phenotypes was observed. Nevertheless, all the patients infected by a GII strain, for whom HBGAs typing was performed, were all *Se*, apart from a single *se*, who was found infected, suggesting a lower likelihood of *se* becoming infected. GI strains belonging to the Lewis-binder group have been described and the single *se* patient was Lewis positive, making it plausible that the GI strain involved in that case belonged to this group. Unfortunately, it was not possible to genotype and amplify the VP1 coding sequence of that GI strain in order to assay its HBGA specificity. Previous studies showed an association with infection and the *Se* phenotype [16]. Nevertheless cases of infected *se* individuals have been described [23]. Consistent with this, several strains belonging to either GI or GII were found capable of binding to carbohydrates present in the *se* individuals, such as the Le^a^ antigen [14], [23].

Since the frequency of *se* individuals in this population is close to 0.2 (data not shown), individuals of this phenotype are under-represented among infected children (1/14). However, this is not statistically significant owing to the small number of cases available.

Genetic sequencing highlighted *FUT2* mutations (*Se^40^* ^, 171,216,357,428,739 and 960^) that have been previously described (BGMUT/NCBI) [50]. There are descriptions of many *FUT2* alleles in the human population, with 19 alleles containing a single substitution (SNP - single nucleotide polymorphisms). Among these, *G428A* is the main inactivating mutation of *FUT2* responsible for the *se* phenotype since it generates a stop codon at position 143 (Trp-X) [51]. This mutation is present in European, African and Iranian populations [52]. In this study, the *Se* phenotype was found in the majority (14/15) of individuals tested. The *se* ^428^ was homozygous in the single individual with a *se* phenotype.

In conclusion, this work revealed that, despite the peculiar characteristics of the Quilombola population under study, rates of NoVs infection in symptomatic and asymptomatic children were consistent with other studies and describes for the first time the circulation of GI.1 and GI.4 in Brazil. Moreover, there was a greater frequency of younger children (<1 year) among the NoV infected children. The preliminary analysis of the genetic diversity of the HBGAs of the Quilombola population revealed a surprising number of similarities with other populations. Although the limited number of positive samples jeopardizes an appropriate analysis, the results showed diverse genotypes of NoV infecting African descendents belonging to different ABO, secretor and Lewis blood groups.

## References

[1] Patel MM, Widdowson MA, Glass RI, Akazawa K, Vinjé J, et al (2008) Systematic literature review of role of norovirus in sporadic gastroenteritis. Emerg Inf Dis. 14: 1224–1231. 10.3201/eid1408.071114 PMC2600393 18680645

[2] Glass RI, Parashar UD, Estes MK (2009) Norovirus gastroenteritis. N Engl J Med. 361: 1776–1785. 10.1056/NEJMra0804575 PMC3880795 19864676

[3] Barreira DMPG, Ferreira MSR, Fumian TM, Checon R, Sadovsky ADI, et al (2010) Viral load and genotypes of noroviruses in symptomatic and asymptomatic children in Southeastern, Brazil. J. Clin. Virol. 47(1): 60–64. 10.1016/j.jcv.2009.11.012 20004146

[4] Desai R, Oliveira LH, Parashar UD, Lopman B, Tate JE, et al (2011) Reduction in morbidity and mortality from childhood diarrhoeal disease after species A rotavirus vaccine introduction in Latin America - a review. Mem. Inst. Oswaldo Cruz (106(8)) 907–911. 10.1590/s0074-02762011000800002 22241109

[5] Trivedi TK, Desai R, Hall AJ, Patel M, Parashar UD, et al.. (2012) Clinical characteristics of norovirus-associated deaths: A systematic literature review. Am J Infect Control. Dec 21. pii: S0196–6553(12)01155–8. doi: 10.1016/j.ajic.2012.08.002 10.1016/j.ajic.2012.08.002 23266383

[6] Hall AJ, Vinjé J, Lopman B, Park GW, Yen C, et al (2011) Center for Disease Control and Prevention. Updated norovirus outbreak management and disease prevention guidelines. MMWR. 60(3): 1–15.

[7] Bennett S, MacLean A, Miller RS, Aitken C, Gunson RN (2013) Increased norovirus activity in Scotland in 2012 is associated with the emergence of a new norovirus GII.4 variant. Euro Surveill. 18(2): pii = 20349. 23324428

[8] Jiang X, Wang M, Graham DY, Estes MK (1992) Expression, self-assembly, and antigenicity of the Norwalk virus capsid protein. J Virol. 66: 6527–6532. 10.1128/jvi.66.11.6527-6532.1992 PMC240146 1328679

[9] Marionneau S, N Ruvöen-Clouet, B Le Moullac-Vaidye, M Clement, A Cailleau-Thomas, et al.. (2002) Norwalk virus binds to histo-blood group antigens on gastro-duodenal epithelial cells of secretor individuals. Gastroenterology 122, 1967–1977. 10.1053/gast.2002.33661 PMC7172544 12055602

[10] Harrington PR, Lindensmith L, Yount B, Moe CL, Baric RS (2002) Binding of Norwalk virus-like particles to ABH histo-blood group antigens is blocked by antisera from infected human volunteers or experimentally vaccinated mice. J Virol. 76: 12325–12343. 10.1128/JVI.76.23.12335-12343.2002 PMC136916 12414974

[11] Huang P, Farkas T, Marionneau S, Zhong W, Ruvoen-Clouet N, et al (2003) Noroviruses bind to human ABO, Lewis, and secretor histo-blood group antigens: identification of 4 distinct strain-specific patterns. J Infect Dis. 188: 19–31. 10.1086/375742 12825167

[12] Nilsson J, Rydell GE, Le Pendu J, Larson G (2009) Norwalk virus-like particles bind to A, H and difucosylated Lewis but not to B histo-blood group active glycosphingolipids. Glicoconj J. 26: 1171–1180. 10.1007/s10719-009-9237-x 19387828

[13] Rydell GE, Nilsson J, Rodriguez-Diaz J, Ruvoën-Clouet N, Svensson L, et al (2009) Human noroviruses recognize sialyl Lewis x neoglycoprotein. Glycobiology 19: 309–320. 19054801 10.1093/glycob/cwn139

[14] Kubota T, Kumagai A, Ito H, Furukawa S, Someya Y, et al (2012) Structural basis for the recognition of Lewis antigens by genogroup I norovirus. J Virol. 86(20: 11138–11150. 10.1128/JVI.00278-12 PMC3457155 22855491

[15] Marionneau S, Cailleau-Thomas A, Rocher J, Le Moullac-Vaidye B, Ruvoën-Clouet B, et al (2001) ABH and Lewis histo-blood group antigens, a model for the meaning of oligosaccharide diversity in the face of a changing world. Biochimie 83: 565–573. 11522384 10.1016/s0300-9084(01)01321-9

[16] Lindesmith L, Moe C, Marionneau S, Ruvoen N, Jiang X, et al (2003) Human susceptibility and resistance to Norwalk virus infection. Nat Med. 9: 548–553. 10.1038/nm860 12692541

[17] Hutson AM, Airaud F, Le Pendu J, Estes MK, Atmar RL (2005) Norwalk virus infection associates with secretor status genotyped from sera. J Med Virol. 77: 116–120. 10.1002/jmv.20423 16032732

[18] Thorven M, Grahn A, Hedlund KO, Johansson H, Wahlfrid C, et al (2005) A homozygous nonsense mutation (428G>A) in the human FUT2 gene provides resistance to symptomatic norovirus (GGII) infections. J Virol. 79: 15351–15355. 10.1128/JVI.79.24.15351-15355.2005 PMC1315998 16306606

[19] Tan M, Jin M, Xie H, Duan Z, Jiang X, Fang Z (2008) Outbreak studies of a GII-3 and a GII.4 norovirus revealed an association between HBGA phenotypes and viral infection. J Med Virol. 80: 1296–1301. 10.1002/jmv.21200 18461617

[20] Huang P, Farkas T, Zhong W, Tan M, Thornton S, et al (2005) Norovirus and histo-blood group antigenic demonstration of a wide spectrum of strain specificities and classification of two major binding groups among multiple binding patterns. J Virol. 79: 6714–6722. 10.1128/JVI.79.11.6714-6722.2005 PMC1112114 15890909

[21] Bucardo F, Kindberg E, Paniagua M, Grahn A, Larson G, et al (2009) Genetic susceptibility to symptomatic norovirus infection in Nicaragua. J Med Virol. 81: 728–735. 10.1002/jmv.21426 19235844

[22] de Rougemont A, Ruvoën-Clouet N, Simon B, Estienney M, Elie-Caille C, et al (2011) Qualitative and quantitative analysis of the binding of GII.4 norovirus variants onto human blood group antigens. J Virol. 85: 4057–4070. 10.1128/JVI.02077-10 PMC3126233 21345963

[23] Nordgren J, Kindberg E, Lindgren P, Matussek A, Svensson L (2010) Norovirus gastroenteritis outbreak with a secretor-independent susceptibility pattern, Sweden. Emerg Inf Dis. 16: 81–87. 10.3201/eid1601.090633 PMC2874438 20031047

[24] Ferrer-Admetlla A, Sikora M, Laayouni H, Esteve A, Roubinet F, et al (2009) A natural history of FUT2 polymorphisms in humans. Mol. Biol. Evol. 26(9): 1993–2003. 10.1093/molbev/msp108 19487333

[25] Aragão GC, Mascarenhas JDP, Kaiano JHL, Lucena MSS, Siqueira JAM, et al (2013) Norovirus Diversity in Diarrheic Children from an African-Descendant Settlement in Belém, Northern Brazil. PLoS ONE 8(2): e56608 doi:10.1371/journal.pone.0056608 23457593 10.1371/journal.pone.0056608 PMC3574080

[26] Grahn A, Elmgren A, Aberg L, Svensson L, Jansson PA, et al (2001) Determination of Lewis FUT3 gene mutations by PCR using sequence-specific primers enables efficient genotyping of clinical simples. Hum Mutat. 18(4): 358–359. 10.1002/humu.1204 11668626

[27] Boom R, Sol CJA, Salimans MMM, Jansen CL, Wertheim-van Dillen PME, et al (1990) Rapid and simple method for purification of nucleic acids. J Clin Microbiol. 28(3): 495–503. 10.1128/jcm.28.3.495-503.1990 PMC269651 1691208

[28] Beuret C, Kohler D, Baumgartner A, Luthi TM (2002) Norwalk-like virus sequences in mineral waters: one-year monitoring of three brands. App Environ Microbiol. 68: 1925–1931. 10.1128/AEM.68.4.1925-1931.2002 PMC123876 11916714

[29] Victoria M, Miagostovich MP, Ferreira MS, Vieira CB, Fioretti JM, et al (2009) Bayesian coalescent inference reveals high evolutionary rates and expansion of Norovirus populations. Infect Genet Evol. 9(5): 927–932. 10.1016/j.meegid.2009.06.014 19559104

[30] Noel JS, Ando T, Leite JP, Green KY, Dingle KE, et al (1997) Correlation of patient immune responses with genetically characterized small round-structured viruses involved in outbreaks of nonbacterial acute gastroenteritis in the United States, 1990 to 1995. J Med Virol. 53: 372–383. 10.1002/(sici)1096-9071(199712)53:4<372::aid-jmv10>3.0.co;2-h 9407386

[31] Kojima S, Kageyama T, Fukushi S, Hoshino FB, Shinohara M, et al (2002) Genogroup-specific PCR primers for detection of Norwalk-like viruses. J Virol Meth. 100: 107–114. 10.1016/s0166-0934(01)00404-9 11742657

[32] Vinjé J, Hamidjaja RA, Sobsey MD (2004) Development and application of a capsid VP1 (region D) based reverse transcription PCR assay for genotyping of genogroup I and II noroviruses. J Virol Meth. 116: 109–117. 10.1016/j.jviromet.2003.11.001 14738976

[33] Abdel-Rahman SZ (1994) Isolation of DNA using salting-out procedure. J Biochem Toxicol. 9: 191–198. 10.1002/jbt.2570090404 7853353

[34] Procter J, Crawford J, Bunce M, Welsh KI (1997) A rapid molecular method (polymerase chain reaction with sequence-specific primers) to genotype for ABO blood group and secretor status and its potential for organ transplants. Tissue Antigens. 50: 475–483. 10.1111/j.1399-0039.1997.tb02902.x 9389321

[35] Marshall JA, Hellard ME, Sinclair MI, Fairley CK, Cox BJ, et al (2003) Incidence and characteristics of endemic Norwalk-like virus-associated gastroenteritis. J Med Virol. 69: 568–578. 10.1002/jmv.10346 12601766

[36] Lau CS, Wong DA, Tong LK, Lo JY, Ma AM, et al (2004) High rate and changing molecular epidemiology pattern of norovirus infections in sporadic cases and outbreaks os gastroenteritis in Hong Kong. J Med Virol. 73: 113–117. 10.1002/jmv.20066 15042657

[37] Monica B, Ramani S, Banerjee I, Primrose B, Iturriza-Gomara M, et al (2007) Human Caliciviruses in Symptomatic and Asymptomatic Infections in Children in Vellore, South India. J Med Virol. 79(5): 544–551. 10.1002/jmv.20862 PMC2473265 17385696

[38] Donaldson EF, Lindesmith LC, LoBue AD, Baric RS (2010) Viral shape-shifting: norovirus evasion of the human immune system. Nat. Rev. Microbiol. 8: 231–241. 10.1038/nrmicro2296 PMC7097584 20125087

[39] Fumian TM, Aragão GC, Mascarenhas JD, Kaiano JH, Siqueira AM, et al (2012) Detection of a novel recombinant strain from the Amazon region of Brazil in 2008. Arch Virol. 157(12): 2389–2392 doi: –––10.1007/s00705–012–1428–2 10.1007/s00705-012-1428-2 22872050

[40] Vega E, Vinjé J (2011) Novel GII.12 Norovirus strains, United States, 2009–2010. Emerg Infect Dis. 17(8): 1516–1518. 10.3201/eid1708.110025 PMC3381559 21801639

[41] Zakikhany K, Allen DJ, Brown D, Iturriza-Gómara M (2012) Molecular Evolution of GII-4 norovirus strains. PLoS ONE 7(7): e41625 doi:10.1371/journal.pone.0041625 22844506 10.1371/journal.pone.0041625 PMC3406047

[42] Ferreira MSR, Xavier MPTP, Tinga ACC, Rose TL, Fumian TM, et al (2012) Assessment of Gastroenteric Viruses Frequency in a Children’s Day Care Center in Rio de Janeiro, Brazil: A Fifteen Year study (1994–2008). PLoS ONE. 7(3): e33754 doi:10.1371/journal.pone.0033754 10.1371/journal.pone.0033754 PMC3309004 22448271

[43] Fioretti JM, Ferreira MSR, Victoria M, Vieira CB, Xavier MPTP, et al (2011) Genetic diversity of noroviruses in Brazil. Mem Inst Oswaldo Cruz. 106(8): 942–947. 10.1590/s0074-02762011000800008 22241115

[44] Soares CC, Santos N, Beard RS, Albuquerque MC, Maranhão AG, et al (2007) Norovirus detection and genotyping for children with gastroenteritis, Brazil. Emerg Infect Dis. 13(8): 1244–1246. 10.3201/eid1308.070300 PMC2828093 17953103

[45] Fukuda S, Takao S, Shigemoto N, Tanizawa Y, Seno M (2010) Transition of genotypes associated with norovirus gastroenteritis outbreaks in a limited area of Japan, Hiroshima Prefecture, during eight epidemic seasons. Arch Virol. 155: 111–115. 10.1007/s00705-009-0528-0 19949962

[46] Lindesmith LC, Donaldson EF, Lobue AD, Cannon JL, Zheng DP, et al (2008) Mechanisms of GII.4 norovirus persistence in human populations. PLoS Med. 5: e31 doi:10.1371/journal.pmed.0050031 10.1371/journal.pmed.0050031 PMC2235898 18271619

[47] Siebenga JJ, Vennema H, Zheng DP, Vinjé J, Lee BE, et al (2009) Norovirus illness is a global problem: emergence and spread of norovirus GII.4 variants, 2001–2007. J Infect Dis. 200: 802–812. 10.1086/605127 19627248

[48] Boon D, Mahar JE, Abente EJ, Kirkwood CD, Purcell RH, et al (2011) Comparative evolution of GII.3 and GII.4 norovirus over a 31-year period. J Virol. 85: 8656–8666. 10.1128/JVI.00472-11 PMC3165818 21715504

[49] Bull RA, White PA (2011) Mechanisms of GII.4 norovirus evolution. Trends in Microbiol. 19(5): 233–240. 10.1016/j.tim.2011.01.002 21310617

[50] Patnaik SK, Helmberg W, Blumenfeld OO (2011) BGMUT: NCBI dbRBC database of allelic variations of genes encoding antigens of blood group systems Nucleic Acids Research. 13: 1–7. 10.1093/nar/gkr958 PMC3245102 22084196

[51] Kelly RJ, Rouqyuer S, Giorgi D, Lennon GG, Lowe JB (1995) Sequence and expression of a candidate for the human secretor blood group alfa (1,2)-fucosyltransferase gene (FUT2). Homozygosity for an enzyme-inactivating nonsense mutation commonly correlates with the non-secretor phenotype. J Biol Chem. 270: 4640–4649. 10.1074/jbc.270.9.4640 7876235

[52] Koda Y, Tachida H, Liu Y, Soejima M, Ghaderi AA, et al (2001) Contrasting patterns of polymorphisms at the ABO-secretor gene (FUT2) and plasma alpha(1,3)-fucosyltransferase gene (FUT6) in human populations. Genetics. 158: 747–756. 10.1093/genetics/158.2.747 PMC1461689 11404338

